# Effect of elicitors on holm oak somatic embryo development and efficacy inducing tolerance to *Phytophthora cinnamomi*

**DOI:** 10.1038/s41598-020-71985-w

**Published:** 2020-09-16

**Authors:** M. Morcillo, E. Sales, L. Ponce, A. Guillén, J. Segura, I. Arrillaga

**Affiliations:** 1 ISIC/ERI BIOTECMED, Departamento Biología Vegetal, Universidad de Valencia, Avda Vicent Andrés Estellés s/n, 46100 Burjassot Valencia, Spain; 2 Departamento de Ciencias Agrarias y del Medio Natural, Escuela Politécnica Superior, Universidad de Zaragoza, Ctra Cuarte s/n, 22071 Huesca, Spain

**Keywords:** Biotechnology, Plant sciences

## Abstract

Holm oak trees (*Quercus ilex* L.) mortality is increasing worryingly in the Mediterranean area in the last years. To a large degree this mortality is caused by the oomycete *Phytophthora* spp., which is responsible for forest decline and dieback in evergreen oak forest areas of the southwestern Iberian Peninsula. This study is based on the possibility of applying chemical elicitors or filtered oomycete extracts to holm oak somatic embryos (SE) in order to induce epigenetic memory, priming, that may increase tolerance to the pathogen in future infections. To this end, we first examined the effect of priming treatments on SE development and its oxidative stress state, to avoid elicitors that may cause damage to embryogenic tissues. Both, the sterile oomycete extracts and the chemical elicitor methyl jasmonate (MeJA) did not produce any detrimental effect on SE growth and development, unlike the elicitors benzothiadiazole (BTH) and *p*-aminobenzoic acid (PABA) that reduced the relative weight gain and resulted in necrotic and deformed SE when were applied at high concentrations (25 µM BTH or 50 µM PABA) in accordance with their high malondialdehyde content. No significant differences among elicitation treatments were found in dual culture bioassays, although those SEs elicited with 50 µM MeJA increased H_2_O_2_ production after challenged against active oomycete indicating the activation of stress response. Since this elicitation treatment did not produce any adverse effect in the embryogenic process we suggest that could be used in further priming experiments to produce holm oak plants adapted to biotic stress.

## Introduction

Holm oak (*Quercus ilex* L.) is one of the most representative evergreen species in natural forest ecosystems of the Mediterranean Basin^1^, covering in Spain around 3 million hectares^2^. This tree species along with cork oak (*Q. suber* L.) dominates the landscape in the agrosilvo pastoral systems (ASPS), called the *dehesa* in Spain and *montado* in Portugal. These ASPS support a diversity of ecosystems services, such as biodiversity, regulation of air and water quality, contribution to soil formation and protection from erosion, and climate change regulation by carbon sequestration^3,4^. In addition, both species are of great economical value, since these trees are sources of cork and acorns, being involved in Iberian pigs extensively raising and also in truffle production from natural woodlands and orchards. These facts could improve rural development, and consequently, allow the stabilization of population in depressed areas^5–7^. These ecosystems also have a great landscape, historical and cultural value^8^, being part of the Special Areas of Conservation defined in EU Council Directive 92/43/EEC.

Holm and cork oaks decline has occurred in the Mediterranean basin since the beginning of the XX century, but mortality of oaks in dehesas has increased from the 80’s onwards. By that time, the soil borne root oomycete *Phytophthora cinnamomi* was often recovered from declining stands, so currently, it is considered the main causal factor associated with this syndrome^9–11^. The beginning of infection requires warm and wet soils, in which *P. cinnamomi* affects the fine feeder roots, while lesions extend to major roots in deeper attacks, and finally the oomycete mycelia can collapse the plant vascular system. The progress of the disease courses with tree symptomatology that includes partial or total leaves desiccation^9,11^. Nowadays, the effects of climate change with increasing periods of drought stress aggravate the pathology caused by *Phytophthora cinnamomi,* and survival is becoming a challenge for the dehesa^12^. In this context and besides prevention and restoration programs, new methods of forest protection have to be implemented^13^. Conventional breeding programs to obtain disease tolerant/resistant plants have been scarce probably because traditional propagation is extremely difficult for the species mainly due to the poor rooting ability of cuttings, which is also reduced with the aging of parent plants^14^. Recently, a holm- and cork-oaks genetic improvement program to alleviate the “seca” syndrome has been launched by the Spanish Government (National Rural Development Program 2014–2020) cofunded by the European Agricultural Fund for Rural Development (EAFRD). Biotechnological tools allowing clonal propagation of selected resistant genotypes such as axillary budding^15^ or somatic embryogenesis^14,16–18^ are available, although acclimazation of generated plants needs to be improved; also, the induction of systemic resistance, triggering natural plant defenses by the use of elicitors, may offer complementary strategies to breeding programs developed to obtain resilient plant material.

Plants respond to oomycete infection by multiple defense mechanisms, including strengthening of physical barriers, production of antimicrobial molecules, and programmed cell death^19^. In addition, many biological and chemical elicitors, such as necrotizing pathogens, salicylic acid (SA), benzothiadiazole (BTH), and methyl jasmonate (MeJA), have been reported to activate plant immune response to pathogens^20–23^. These elicitors cause the induction of a primed state of enhanced defenses which allows plants to display either faster, stronger, or both, activation of the defense responses after further attacks by pathogens or insects, and also under abiotic stress conditions^24^. Thus, exogenous applications of SA and carvacrol to *Ulmus minor* successfully enhanced the resistance of trees to the fungal pathogen *Ophiostoma novo-ulmi*^25,26^. Also, foliar sprays of SA or BTH to *Pinus radiata* significantly decreased plant infections by *Diplodia pinea*^27^ or by *Phytophthora cinnamomi*^28^. Primed plants present changes at the physiological, molecular, and epigenetic levels that can occur within seconds or hours after stimulation; these changes can be transient or maintained for its lifetime, and can even be inherited by subsequent generations^29^. The most common molecular mechanisms by which a plant is primed involve chromatin modifications, either DNA methylation, histones changes or posttranscriptional variations, which are the basis of inheritable regulation in the expression and gene function that does not involve DNA sequence alterations but epigenetic variations^30^. There are some evidences that biotic stresses exerted on mother plants by herbivores or pathogens can induce transgenerational defenses in progeny when challenging environmental harshness^31,32^. However, most studies of transgenerational induction of defenses to pests and pathogens in plants have focused on short-lived annuals. As an example, applications of β-amino-butyric acid (BABA) as well as virulent or avirulent strains of *Pseudomonas syringae* pv. *tomato* to arabidopsis plants displayed enhanced disease resistance in their progenies when were infected with this phytopathogenic bacteria or with the biotrophic oomycete *Hyaloperonospora arabidopsidis*^33,34^. However, it remains largely unknown whether this type of transgenerational plasticity also occurs in long-lived forest trees ^31^. Environmental maternal effects on the tolerance of *Pinus pinaster* to biotic stress have been reported^32^. In addition, a recent work^35^, demonstrated that chestnut (*Castanea sativa*) seedlings of ink-diseased mother trees showed increased tolerance to *P. cinnamomi*; interestingly, this tolerance was not mediated by seed size, but probably as a consequence of seed priming during fruit development. These inheritable variations could open doors for obtaining resistant or tolerant oak genotypes, which would be included in a recovery program in damaged areas, avoiding oak woodlands and *dehesa* dieback.

Since somatic embryo (SE) development mimics zygotic embryogenesis, we hypothesize that priming SEs during the proliferation phase may induce transgenerational defense mechanisms that would produce resistance to *P. cinnamomi* in subsequent infections. It is of practical interest to determine first if elicitor molecules released during the early stages of the plant–pathogen interaction could be directly applied to embryogenic lines without altering their development, and second, whether these elicitors may activate priming signals in order to suppress the deleterious effects of fungal diseases in plants. Because of this, the objectives of this work were to assay the effect of some elicitors on holm oak SE development, and to study the preliminary responses of elicited SEs after infection with *P. cinnamomi.*

## Results

### Effect of *Phytophthora* filtered extract (PFE) and chemical elicitors on holm oak SE growth and development

The transference of elicited material to proliferation medium produced secondary embryogenesis and some of the globular embryos underwent maturation up to the early cotyledonary stage. Some elicited-SEs turned necrotic and therefore were subtracted from total well-formed embryos.

**Figure 1:**
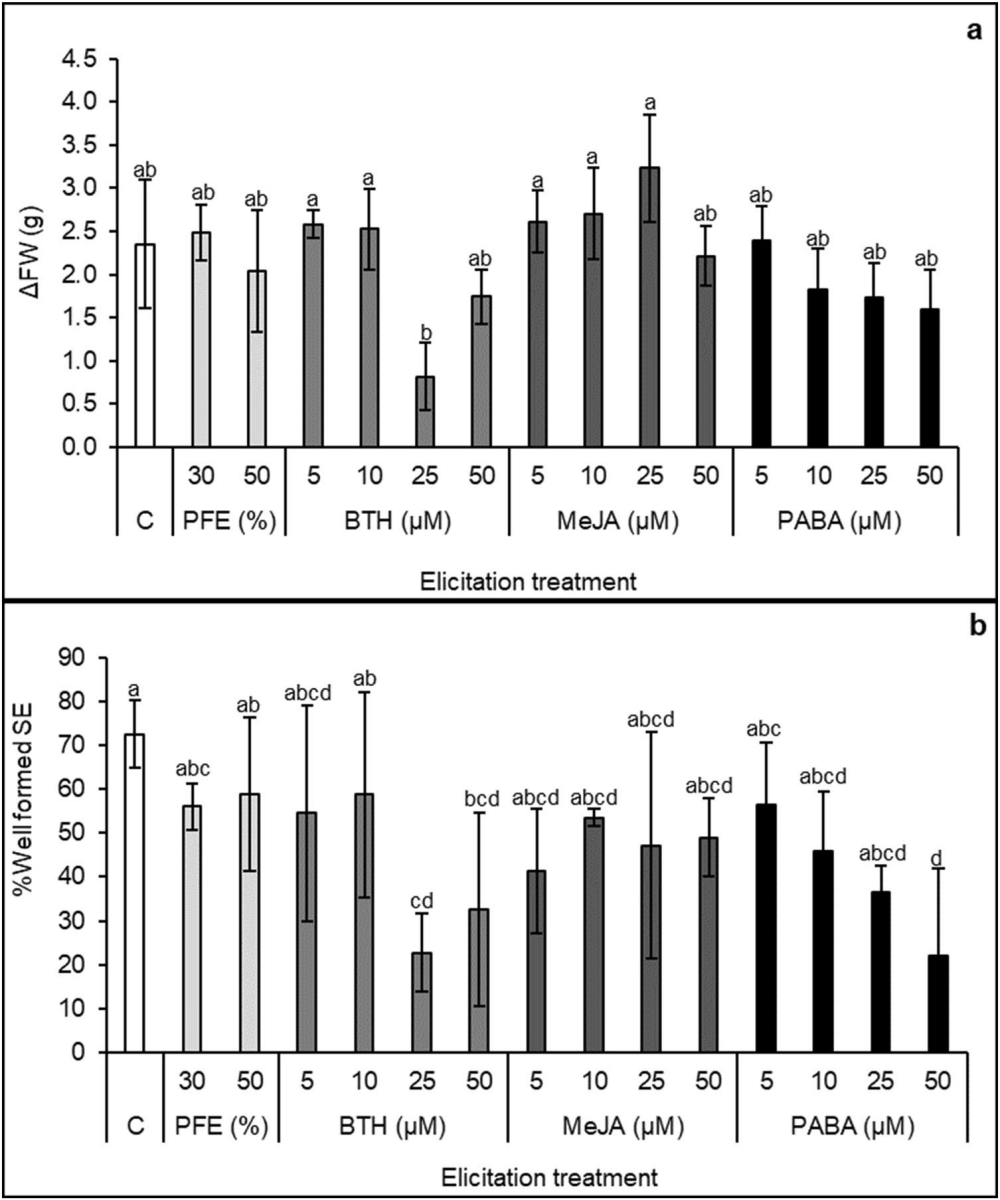
Effect of *Phytophthora* Filtered Extract, PFE, diluted at 30 or 50% and benzothiadiazole (BTH), methyl jasmonate (MeJA) and *p*-aminobenzoic acid (PABA) at 5, 10, 25 and 50 µM, on holm oak somatic embryo development on (**a**) increment of fresh weight; and (**b**) percentage of well-developed embryos. C (control treatment). Data are mean ± SD of three replicates. In (**a**) values followed by the same letter were not different according to Tukey-B test.

After 60 days of elicitation significant differences in SE growth (*p* < 0.001) among elicitation treatments were found. Fresh weight increase was higher on globular SE elicited with MeJA at 5, 10 or 25 µM, as well as for those elicited with 5 or 10 µM BTH, but reduced on those SEs elicited with 25 µM BTH (Fig. 1a). However, SE growth rates achieved in these treatments did not significantly differ to that of controls (2.4 ± 0.9 g), although on average, we observed higher growth rate for MeJA-elicited SEs (2.7 ± 0.6 g) as compared to BTH and PABA treatments (means 1.9 ± 0.8 and 1.9 ± 0.6 g, respectively, Fig. 1a) and these differences were statistically significant (*p* = 0.01). Growth rate of PFE-elicited SEs was on average similar to that of non-elicited embryogenic material (2.3 ± 0.7 g, Fig. 1a).

The analysis of variance showed significant differences on holm oak somatic embryo development among the 14 elicitation treatments tested (*p* < 0.001, Fig. 1b). On average, PABA-elicitation treatments significantly reduced the percentage of well-developed embryos as compared to controls (40.2 ± 19.1% vs. 72.5 ± 7.8%, *p* = 0.047), particularly at the highest concentration (50 µM). This detrimental effect was also observed for BTH-elicitation treatments at 25 and 50 µM. In contrast, PFE-elicited embryogenic material produced, on average, similar rates of well-developed SEs (57.4 ± 13.0%) to that of controls, as occurred with MeJA treatments (47.7 ± 16.0%). These results would indicate a more detrimental effect on holm oak SEs development of BTH and PABA elicitation treatments, especially when higher concentrations of these compounds are used, as compared to application of MeJA or PFE.

### Effect of elicitation treatments on MDA content of holm oak SEs

**Figure 2:**
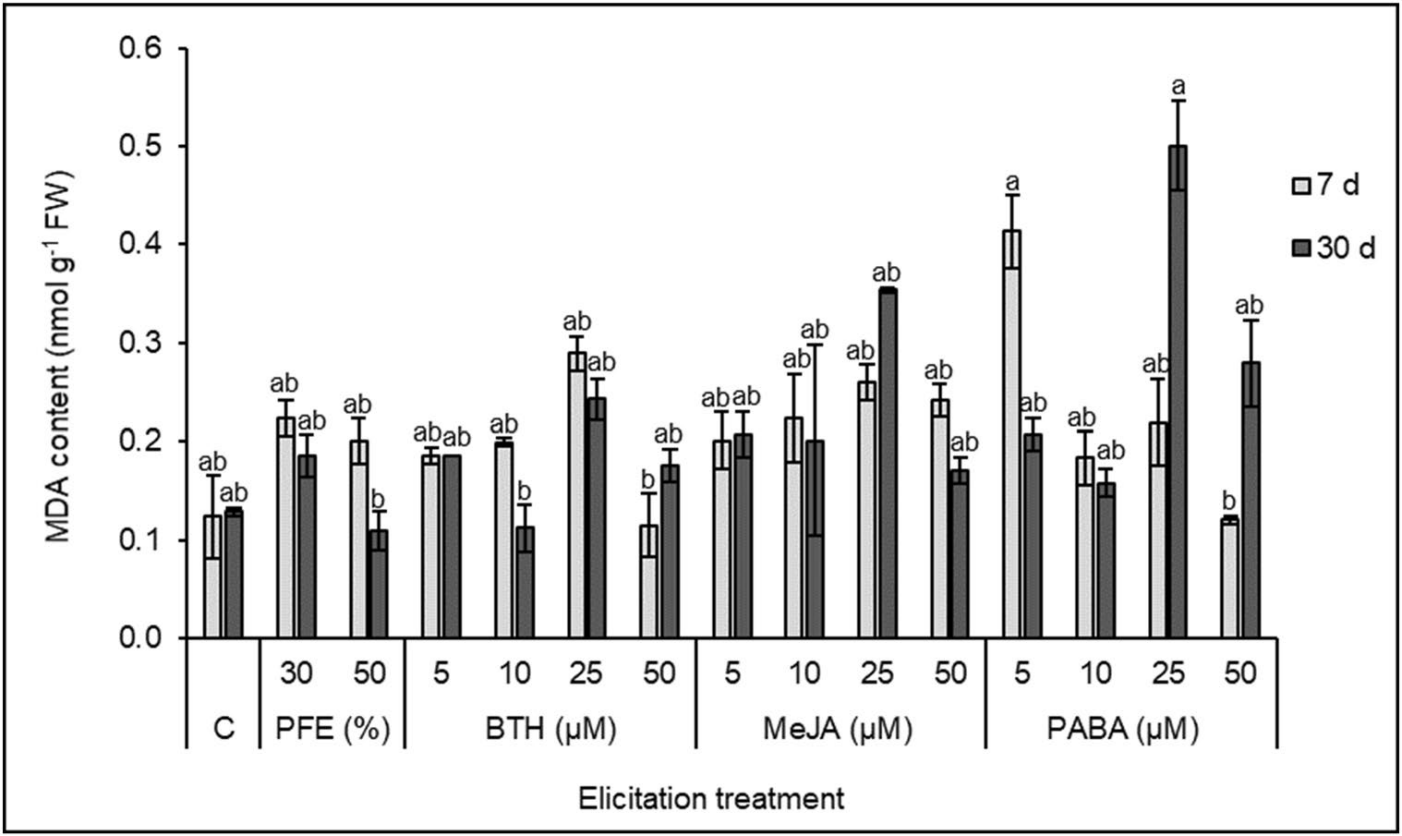
MDA content (nmol g^−1^ FW) in holm oak somatic embryos after 7 and 30 days of control (C) or elicitation treatments with *Phytophthora* filtered extract (PFE), diluted at 30 or 50%, or benzothiadiazole (BTH), methyl jasmonate (MeJA) or *p*-aminobenzoic acid (PABA) at 5, 10, 25 or 50 µM. Data are mean ± SD of three replicates. Within each time, values followed by the same letter were not different according to Kruskal–Wallis test with Bonferroni correction for multiple comparisons.

Irrespective of the elicitation treatment (biological or chemical), levels of MDA in holm oak SEs slightly increased 7d after elicitation (Fig. 2). Nevertheless, analysis performed by Kruskal–Wallis non-parametric test found significant differences only for SEs elicited with PABA at 5 µM, where MDA content increased from 0.124 ± 0.042 nmol g^−1^ FW in non-elicited material to 0.413 ± 0.038 nmol g^−1^ FW, while treatments at the highest BTH and PABA concentration (50 µM), showed the lower MDA levels (0.115 ± 0.033 and 0.120 ± 0.005 nmol g^−1^ FW, respectively). Note that 30 days after elicitation, MDA levels had recovered in SEs elicited with 5 µM PABA (0.206 ± 0.017 nmol g^−1^ FW, which are closer to 0.129 ± 0.004 nmol g^−1^ FW in controls), but increased in those elicited with 25 µM or 50 µM PABA (from 0.219 ± 0.044 to 0.501 ± 0.046 nmol g^−1^ FW and from 0.120 ± 0.005 to 0.279 ± 0.044 nmol g^−1^ FW, respectively, Fig. 2). The high MDA content observed in SEs elicited with 25 µM PABA 30 d after the treatment contrasted to the lower levels detected in SEs elicited with either the oomycete extract PFE-50 (0.109 ± 0.020 nmol g^−1^ FW) or 10 µM BTH (0.112 ± 0.024 nmol g^−1^ FW).

### *Phytophthora cinnamomi* growth in dual cultures with elicited holm oak SEs

**Figure 3:**
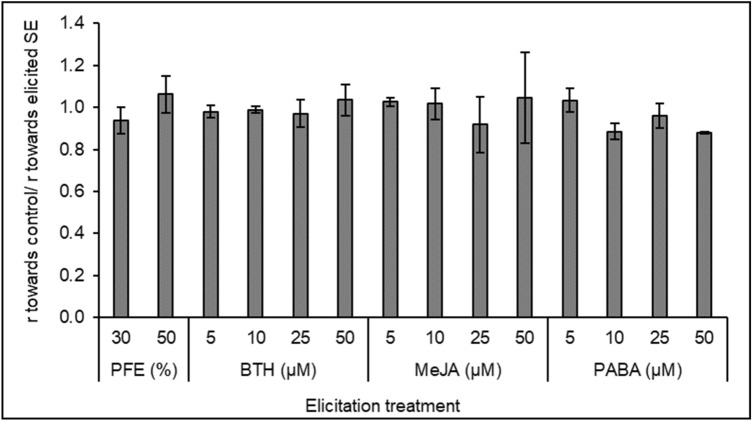
Dual cultures: mean *Phytophthora cinnamomi* mycelia growth ratios (towards control/elicited) measured after 3 days of challenging control and elicited embryogenic material with active oomycete. Elicitation treatments were performed in triplicate using *Phytophthora* filtered extract (PFE) diluted at 30 or 50% or benzothiadiazole (BTH), methyl jasmonate (MeJA) or *p*-aminobenzoic acid (PABA) at 5, 10, 25 or 50 µM.

When *Phytophthora cinnamomi* mycelia were inoculated in plates containing control and elicited holm oak globular SEs, similar hyphae growth rates were observed towards both types of material, (ratios r_control_/r_elicited_ around 1). As it is shown in Fig. 3, at the end of the experiment (day 3) a significant inhibitory effect of elicited SEs was not detected in these bioassays.

### Hydrogen peroxide production in holm oak SEs after *Phytophthora cinnamomi* dual cultures

**Figure 4:**
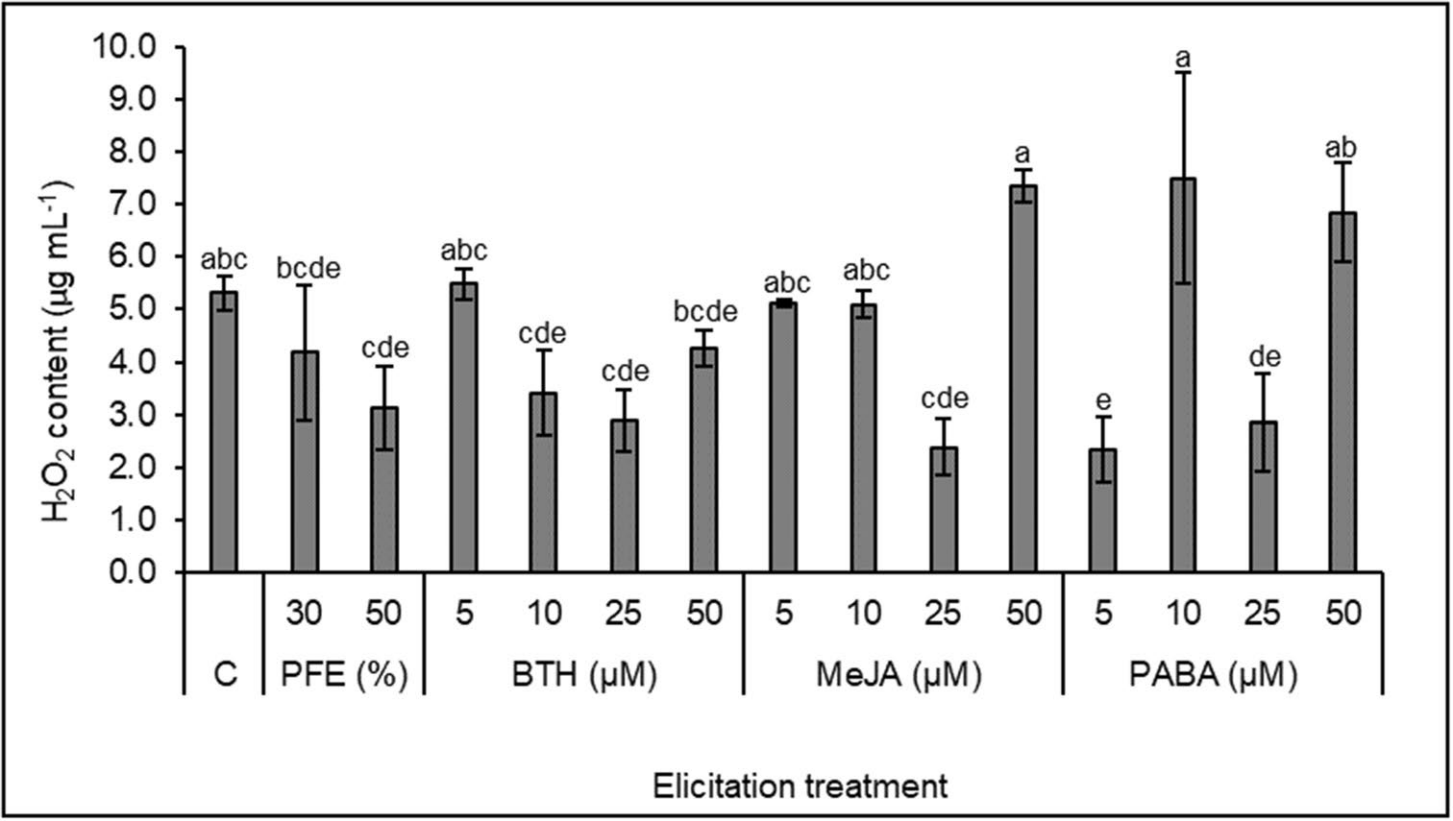
Hydrogen peroxide content (µg mL^−1^) in control (C) and elicited holm oak somatic embryos after dual cultures with *Phytophthora cinnamomi*. Elicitation treatments tested were *Phytophthora* filtered extract (PFE) diluted at 30 or 50%, or benzothiadiazole (BTH), methyl jasmonate (MeJA) or *p*-aminobenzoic acid (PABA) at 5, 10, 25 or 50 µM. Values are mean ± SD of three replicates, and those followed by the same letter were not significantly different according to Tukey-B test.

Hydrogen peroxide production in control and elicited holm oak SEs was determined after challenging this material with oomycete mycelia in dual culture assays. As shown in Fig. 4, significant differences in H_2_O_2_ production were observed among samples from different elicitation treatments. Higher H_2_O_2_ level in response to oomycete challenge was determined in SEs elicited with 50 µM MeJA (7.35 ± 0.32 µg mL^−1^) or 10 µM PABA (7.50 ± 2.00 µg mL^−1^), although values did not differ significantly from that found in non-elicited material. In contrast, H_2_O_2_ production was significantly repressed in holm oak SEs elicited with 5 or 25 µM PABA as compared to controls (2.34 ± 0.61 and 2.86 ± 0.92 µg mL^−1^, respectively, vs 5.31 ± 0.33 µg mL^−1^).

## Discussion

The design of an effective strategy to induce tolerance to *P. cinnamomi* in holm oak must consider the effect of elicitation treatments on the embryogenic material, to avoid a decrease in somatic embryos proliferation that impede the plant regeneration^36^. In our study, abnormally developed embryos were mainly observed in embryogenic material elicited with high concentrations (25 or 50 µM) of BTH or PABA. Other authors also reported that high concentrations of some elicitors could negatively influence the biologic development of plant material^37^. On the contrary, MeJA application produced a recovery in the differentiation capacity of an embryogenic line of *Quercus ilex*^36^. Then, possible detrimental effects of the elicitation treatments tested herein were also assessed by determining MDA content of holm oak SEs. MDA production indicates high lipid peroxidation in plant cells^38–41^. In our experiments this compound was detected in higher concentration in SEs from all elicitation treatments, although MDA contents were not significantly different from that of controls. Interestingly, 7 d after elicitation we observed lower MDA levels in SEs treated with the highest BTH or PABA concentration (50 µM), in contrast to the higher MDA content determined in material elicited with 5 µM PABA. However, after 30 days we observed a recovery of cell membranes integrity in this material, and also in those elicited with PFE or MeJA at the higher concentrations, while MDA content increased in material elicited with 25 or 50 µM PABA or with 50 µM BTH. These results suggest a negative effect of PABA-elicitation on cell membrane integrity of holm oak SEs that could be related to the lower embryo development frequencies observed on average in these treatments. Chong et al.^42^ suggested that damages in cell membranes during the first days after elicitation of *Morinda elliptica* cell cultures with MeJA would trigger the biosynthesis of antioxidant enzymes, therefore inducing a subsequent decrease in MDA.

Experiments using dual cultures have shown that the stimulation or inhibition of mycelium growth in these conditions could give useful information about plant susceptibility^43–45^, and therefore could be used to select efficient elicitation treatments^36,42^. In our study, elicited holm oak somatic embryos did not inhibit *Phytophthora cinnamomi* mycelium growth in dual culture tests, being the plates with somatic embryos elicited with PFE-50, 50 µM BTH, 5, 10 or 50 µM MeJA and 5 µM PABA those showing ratios higher than 1 between growth towards control and growth towards elicited material at the end of the experiment. Treatments with cinnamomin have been reported to inhibit oomycete root colonization in holm oak^21^; foliar applications of a BTH derivate also reduced the incidence of oomycete root infection in several forest^28^; MeJA have been referred to induce resistance in conifers seedlings to a pathogenic oomycete^46^ and pests^47–49^, as well as resistance to pathogens in plants such as arabidopsis^50^, grapevine^51^ or tomato^37^, and in harvested fruits as bayberries^52^ and grapes^53,54^.

Wang et al.^54^ reported that induced resistance to *Botrytis cinerea* of grape fruits treated with MeJA was tightly associated with increased H_2_O_2_ production in this plant material. This biotic stress response was characterized in holm oak elicited somatic embryos, and we found higher H_2_O_2_ contents in samples elicited with 50 µM MeJA and 10 or 50 µM PABA (*p* > 0.05), therefore indicating a better activation of defense mechanism in these plant materials.

In spite of significant differences in all parameters were not found, an overview of our results concerning SE growth and development, MDA production, inhibition of mycelia growth and H_2_O_2_ production suggest that in our conditions 50 µM MeJA seems to be the most adequate elicitation treatment in holm oak embryogenic material. These results contribute to the scarce available information about elicitation of somatic embryogenic material of *Quercus* species.

## Material and methods

### Plant and oomycete material

**Figure 5:**
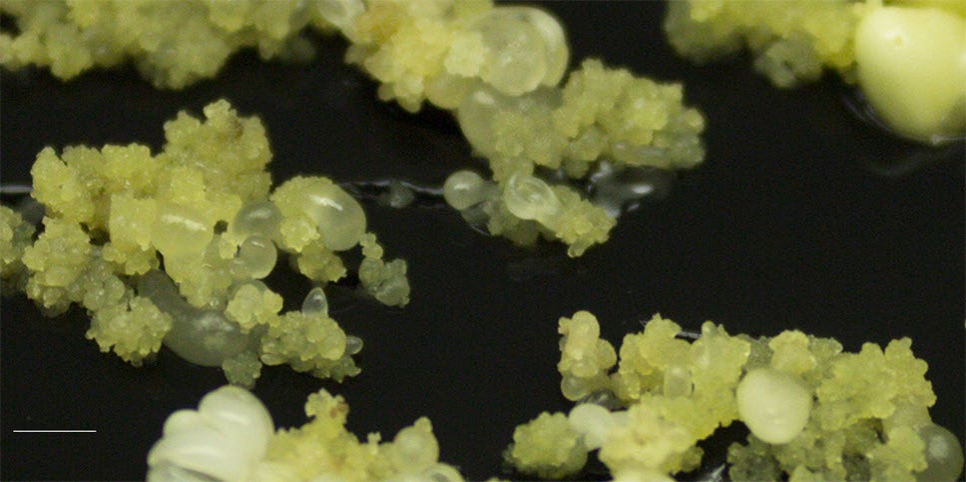
Holm oak embryogenic line Ha13 showing SEs at different developmental stages. Bar 1 cm.

The experiments were undertaken with the embryogenic holm oak (*Quercus ilex* L.) line Ha13. This line was obtained from male catkins of a 50–100-year-old holm oak located in La Hunde mountain (Ayora, Valencia, Spain) as described in^16^ and has being maintained in vitro by monthly subcultures on proliferation MS^55^ medium supplemented with 20 µM silver thiosulfate and 4 g L^−1^ activated charcoal (named after MS/STS/AC medium as described in^56^). On this medium, Ha13 line produces secondary embryogenesis and somatic embryos (SEs) at different developmental stages (Fig. 5) from which material consisting of embryogenic calli and globular SEs was employed for elicitation experiments.

**Figure 6:**
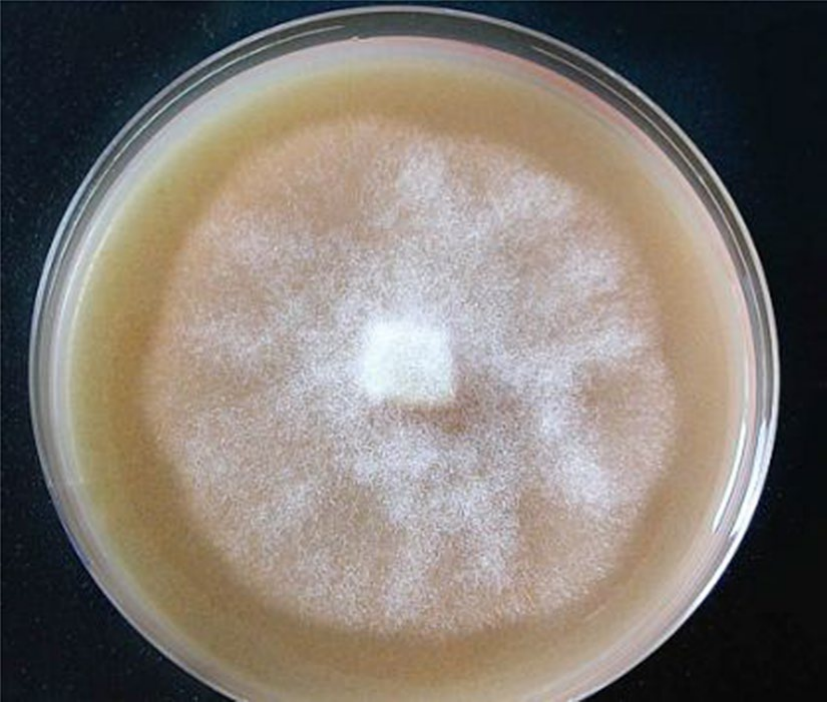
*Phytophthora cinnamomi* 1630 strain after 10 days of transference to fresh PDA medium.

*Phytophthora cinnamomi* strain 1630 was kindly provided by Paloma Abad (group Phytopathogenic fungi, Instituto Agroforestal Mediterráneo—Universidad Politécnica de Valencia, Spain) and was maintained in PDA medium (Potato Dextrose Agar, Pronadisa) by subculturing mycelium pieces of 0.5 cm^2^ to fresh medium every 15 days (Fig. 6).

### Elicitation assays

Elicitation assays were performed with either filtered extract of *Phytophthora cinnamomi* 1630 strain (Fig. 6) diluted at 30 or 50% v/v (named after PFE-30 and PFE-50, respectively), or the chemical elicitors methyl jasmonate (MeJA), benzothiadiazole (BTH) and p-aminobenzoic acid (PABA) at 5, 10, 25 and 50 µM.

To prepare the oomycete extract, flasks containing 40 mL liquid ESM medium (Elicitin Secretion Medium^57^) were inoculated with eight 0.5 cm^2^ *P. cinnamomi* mycelium cuts from outer edge of 1630 strain *P. cinnamomi* mycelium that had been growing in PDA medium, at 25 °C and darkness during 10 days. ESM medium consisted of 500 mg L^−1^ KH_2_PO_4_, 250 mg L^−1^ MgSO_4_·7H_2_O, 1,000 mg L^−1^ asparagine, 1 mg L^−1^ thiamine, 20 mg L^−1^ glucose and 500 mg L^−1^ yeast extract. The oomycete cultures were maintained at 25 °C, in darkness and agitation at 50 rpm for 5 days. After this period, the cultures were collected by filtration using Büchner funnels and 90 mm Whatman filter paper; subsequently, the liquid extracts were sterilized through 0.22 µm Whatman filters^58^; finally, the sterile extract was diluted at 30 or 50% v/v in ESM medium.

For elicitation, the embryogenic line containing SE at the globular stage was cultured for 3 days in 40 mL ESM medium supplemented with the above-mentioned elicitors in agitation at 100 rpm, 25 °C and darkness; after this period, the embryos were recovered on Whatman filter paper, using Büchner funnels, and transferred to MS/STS/AC proliferation medium. The effect of elicitation treatments on holm oak embryogenesis was determined as SE growth (fresh weight increase) and SE development (percentage of well-formed immature cotyledonary SEs) 60 days after elicitation in triplicate.

### Determination of malondialdehyde

To assess the potential damage of elicitors on cell membrane integrity, we determined malondialdehyde (MDA) concentration in control and elicited embryos 7 and 30 days after elicitation, as described in^59^. To this end, 0.1 g of embryogenic material was homogenized in 1 mL of 50 mM extraction solution (0.07% NaH_2_PO_4_·2H_2_O and 1.6% Na_2_HPO_4_·12H_2_O) and centrifuged at 13,000 rpm for 25 min at 4 °C. After that, 200 µL of the supernatant were added to 800 μL of a solution containing 20% trichloroacetic acid (TCA) and 0.5% thiobarbituric acid (TBA) (both from Sigma-Aldrich, USA). The mixture was heated at 95 °C for 30 min, quickly cooled in an ice bath and centrifuged at 13,000 rpm for 10 min. The absorbance of the supernatant was read at 532 and at 600 nm in an Eppendorf BioSpectrometer. The value for the non-specific absorption at 600 nm was subtracted from the reading at 532 nm. MDA concentration was calculated using the MDA extinction coefficient of 155 mM^−1^ cm^−1^ according to^60^. Each treatment was tested in triplicate.

### Growth of *Phytophthora cinnamomi* in dual cultures with elicited holm oak SEs

**Figure 7:**
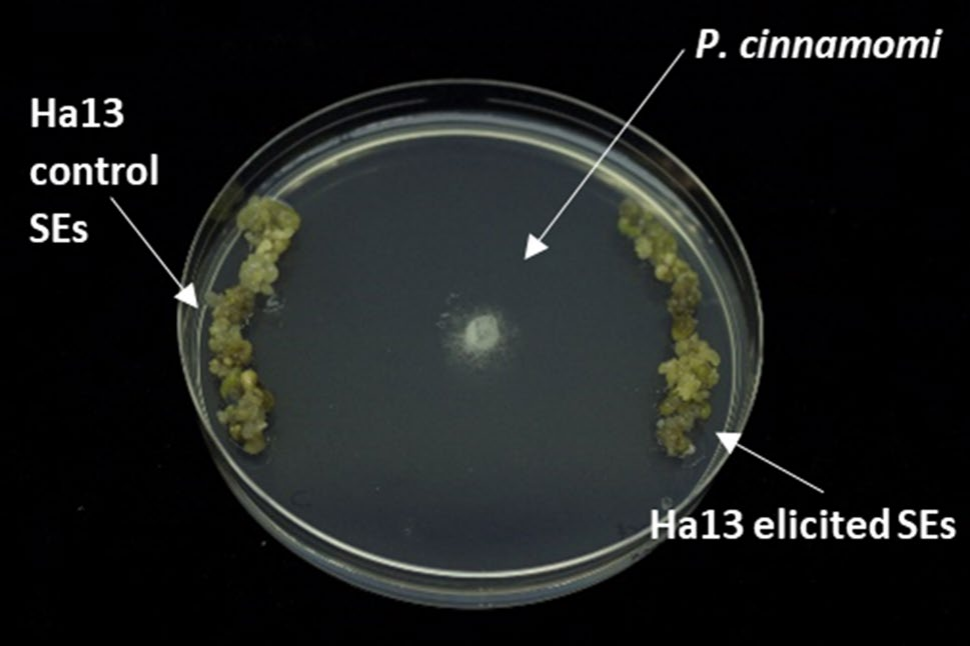
Establishment of dual cultures with *Phytophthora cinnamomi* (middle) and holm oak embryogenic line Ha13 control (left) and elicited (right).

Globular SEs (0.3 g) from elicited and control material maintained in MS/STS/AC medium for 90 days were incubated in the presence of *P. cinnamomi* mycelium (0.5 cm^2^) on 90 mm petri dishes containing SH medium^61^ without plant growth regulators. The section of *P. cinnamomi* mycelium was placed in the middle of the dish (Fig. 7) and the ratio of mycelium growth towards the control and the elicited material was evaluated daily until oomycete hyphae reached SEs (3 days). Therefore, mycelia growth ratios > 1 indicate that the oomycete mycelium spread faster to control samples than to elicited material. Material from each elicitation treatment was tested in triplicate.

### Determination of hydrogen peroxide in plant material during dual cultures

As a measure of primary defense response to oomycete’s infection, we determined hydrogen peroxide (H_2_O_2_) formation in both control and elicited SEs after 3 days of dual culture with *P. cinnamomi* mycelium, according to^42^. Briefly, 0.1 g of elicited or control and infected SEs was homogenized in 1 mL 0.1% (w/v) TCA. The homogenate was centrifuged at 13,000 rpm at 4 °C for 15 min and 250 µL of the supernatant was added to 250 µL 10 mM potassium phosphate buffer (pH 7.0) and 500 µL 1 M potassium iodide. Absorbance of each sample was read at 390 nm and treatments were tested in triplicate.

### Statistical analysis

Data were analyzed by one-way analysis of variance (ANOVA), and are presented as mean ± standard deviation of three independent replications. When appropriate, treatment means were separated using Tukey’s HSD (honestly significant difference^62^). Data that not followed a normal distribution were analyzed by the Kruskal–Wallis one-way non-parametric test (k independent samples) or U-Mann–Whitney test (2 independent samples). The arcsine transformation was applied to percentage data prior to analysis. All statistical analyses were performed using SPSS for Windows, version 26 (SPSS Inc., Chicago, IL, USA).
